# Supplementary figures and images for: Ferroptosis-Related Genes as Molecular Markers in Bovine Mammary Epithelial Cells Challenged with Staphylococcus aureus
Source: Int J Mol Sci. 2025 Mar 11;26(6):2506. doi: 10.3390/ijms26062506 (PMC11942541; doi:10.3390/ijms26062506)

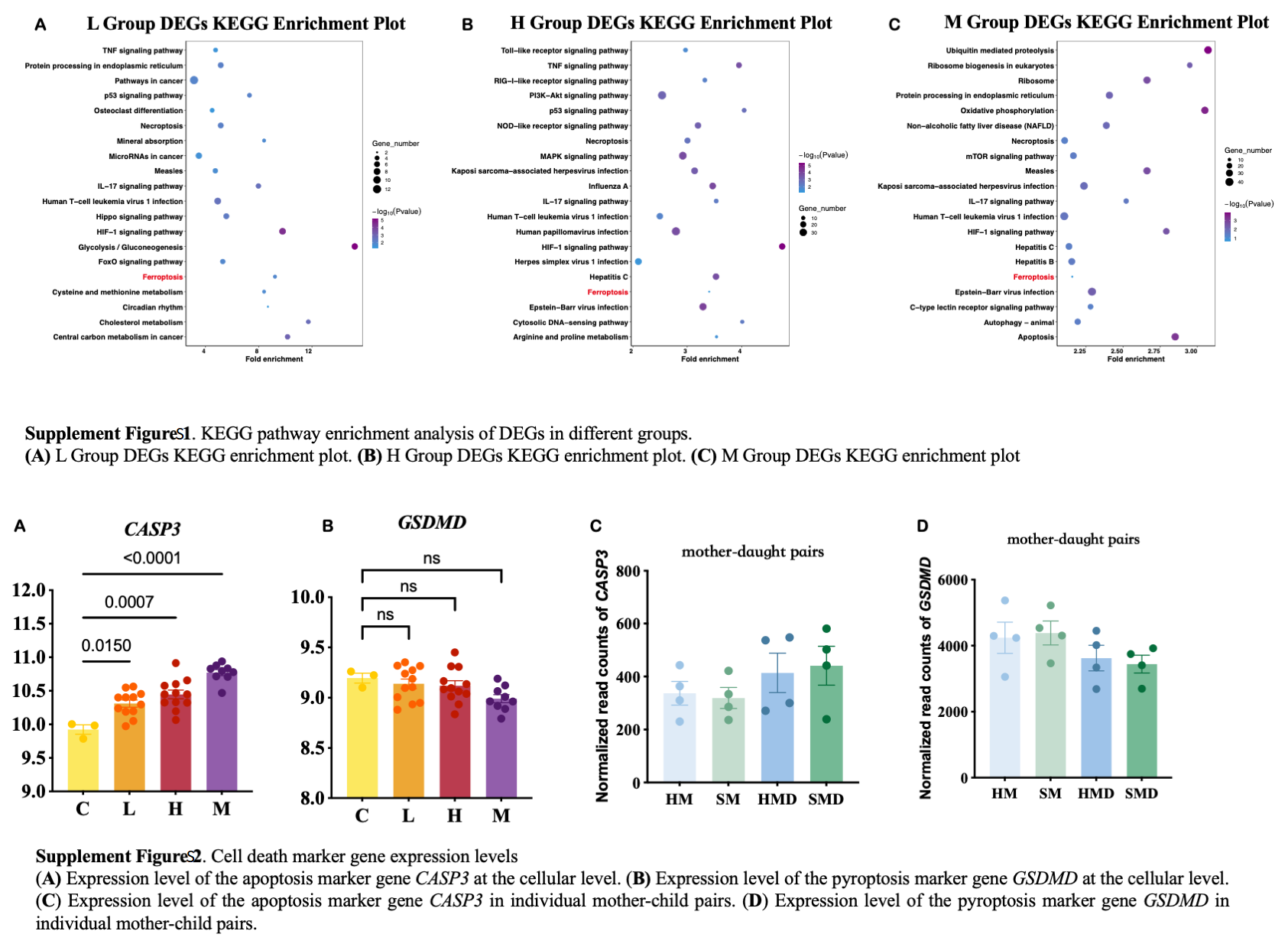

Supplement: Supplementary file 1 [file ijms-26-02506-s001.zip › Figure S1-S2.tiff]

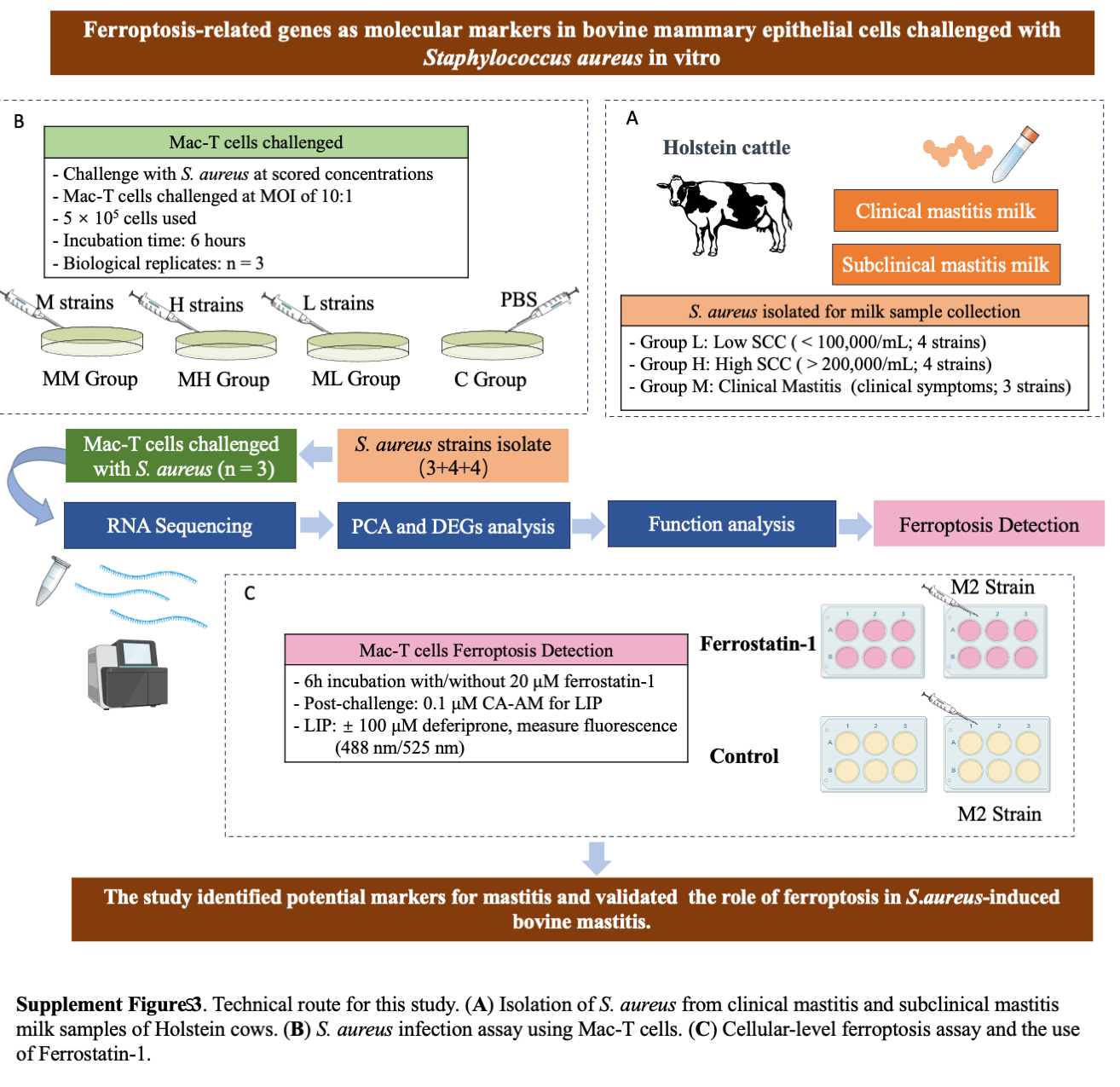

Supplement: Supplementary file 1 [file ijms-26-02506-s001.zip › Figure S3.tiff]
